# Expression and clinical significance of annexin A2 and human epididymis protein 4 in endometrial carcinoma

**DOI:** 10.1186/s13046-015-0208-8

**Published:** 2015-09-11

**Authors:** Lu Deng, Yiping Gao, Xiao Li, Mingbo Cai, Huimin Wang, Huiyu Zhuang, Mingzi Tan, Shuice Liu, Yingying Hao, Bei Lin

**Affiliations:** Department of Gynaecology and Obstetrics, Shengjing Hospital affiliated to China Medical University, No. 36 Sanhao Street, Heping District, Shenyang, 110004 Liaoning China; Tianjin Central Hospital of Gynaecology and Obstetrics, Tianjin, China; Department of Obstetrics and Gynecology, Hospital of Zhengzhou University, Zhengzhou, Henan China; Department of Gynecology and Obstetrics, Beijing chaoyang Hospital, Capital Medical University, Beijing, China

**Keywords:** Annexin A2, Human epididymis protein 4, Endometrial carcinoma, Immunohistochemistry, Prognosis

## Abstract

**Background:**

It is well-known that the treatment and monitoring methods are limited for advanced stage of endometrial carcinoma. Biological molecules with expression changes during tumor progression become potential therapeutic targets for advanced stage endometrial carcinoma. Annexin A2 (ANXA2) has been reported to be overexpressed in recurrent endometrial carcinoma, and the expression of human epididymis protein 4 (HE4) is upregulated in endometrial carcinoma. What’s more, ANXA2 and HE4 interacted in ovarian cancer and promoted the malignant biological behavior. We speculated that their interaction may exist in endometrial carcinoma as well. We evaluated the expression and the correlation relationship of ANXA2 and HE4 in endometrial carcinoma.

**Methods:**

The expression of ANXA2 and HE4 protein in 84 endometrial carcinoma, 30 endometrial atypical hyperplasia, and 18 normal endometrial tissue samples were then measured using an immunohistochemical assay in paraffin embedded endometrial tissues. The structural relationship between ANXA2 and HE4 was explored by immunoprecipitation and double immunofluorescent staining.

**Results:**

ANXA2 and HE4 co-localized in both endometrial tissues and endometrial carcinoma cells. ANXA2 and HE4 were expressed in 95.2 % and 85.7 % of the the endometrial carcinoma, respectively, which were significantly higher than normal endometrium (55.6 % and 16.7 %, both *p* < 0.05). The expression of ANXA2 and HE4 was significantly correlated with FIGO stage, degree of differentiation, myometrial invasion, and lymph node metastasis. ANXA2 was an independent risk factor for the prognosis of endometrial carcinoma (*p* < 0.05, hazard ratio [HR] = 8.004). The expression of ANXA2 and HE4 was positively correlated (Spearman correlation coefficient = 0.228, *p* < 0.05). HE4 was an independent factor for ANXA2 in multivariate linear regression model (*p* < 0.05).

**Conclusion:**

We revealed the co-localization of ANXA2 and HE4 in endometrial carcinoma. Expression levels of ANXA2 and HE4 were closely related to the malignant biological behavior of endometrial carcinoma, and ANXA2 was an independent risk factor for poor prognosis. The expression of ANXA2 and HE4 can affect each other.

**Electronic supplementary material:**

The online version of this article (doi:10.1186/s13046-015-0208-8) contains supplementary material, which is available to authorized users.

## Background

Endometrial carcinoma is one of the most common malignant tumors of the female reproductive system, the mortality of which is only second to ovarian carcinoma in developed countries; the incidence of endometrial carcinoma is increasing year-by-year [1]. Endometrial carcinoma most often affects postmenopausal women, the incidence among younger women has been on the rise in recent years. Many patients with endometrial carcinoma are diagnosed early due to irregular vaginal bleeding, and usually have a good prognosis. Treatment options are limited for patients with metastases in advanced stages or recurrent endometrial carcinoma; there is no significant effect using combined chemotherapy and radiotherapy or novel molecular-targeted drugs [2, 3]. The known prognostic factors of endometrial carcinoma include degree of tumor differentiation, International Federation of Gynecology and Obstetrics (FIGO) stage, histologic type, and estrogen receptor (ER) positivity [4–6], but do not predict patient outcome or guide treatment. Biological molecules with expression changes during tumor progression become potential therapeutic targets for advanced stage endometrial carcinoma. Researchers have identified a large number of new biomarkers that can be used to evaluate the prognosis of endometrial carcinoma and monitor recurrences [7–12], and some molecules associated to the tumorigenesis of endometrial carcinoma [13]; however, no biomarker has been used in clinical application.

Annexin A2 (ANXA2) is a member of the annexin family, and is also known as p36. ANXA2 has a relative molecular mass of 36 kDa and the gene is located on the long arm of chromosome 15 [14]. ANXA2 exists in many cell types and regulates a number of biological functions, including membrane transport, signal transduction, cell differentiation, and apoptosis [15]. A large number of studies have shown a significant change in the level of ANXA2 expression in tumor tissues [16]. The expression of ANXA2 is significantly increased in the malignant tumors of the blood, and breast, cervical, and pancreatic cancers, and is also related to drug resistance and poor prognosis. The expression of ANXA2 is decreased in esophageal squamous cell carcinoma and osteosarcomas, while the change of expression in prostate cancer is controversial [16, 17]. ANXA2 is closely related to the occurrence and development of tumors, and also plays an important role in angiogenesis, proliferation, apoptosis, adhesion, invasion, and migration in malignant tumors [18].

Human epididymis protein 4 (HE4), also known as whey acidic protein (WFDC2), is an ovarian cancer tumor marker identified by genomic and proteomic screening [19]. HE4 was designated as a serum marker for ovarian cancer in 2003, and aroused the attention of researchers due to its high sensitivity and specificity [20]. HE4 is a secreted protein, with a much smaller molecular weight compared to CA-125, and has a better sensitivity, specificity, positive likelihood ratio, and negative likelihood ratio than CA-125 in the diagnosis of ovarian cancer [21]. Subsequent research has shown that the overexpression of HE4 not only exists in patients with ovarian cancer, but has also been observed in patients with transitional cell carcinoma, lung adenocarcinoma, breast cancer, pancreatic cancer, endometrial carcinoma and so on [22, 23].

In the preliminary study, a known protein annexin a2 was discovered as a binding partner of HE4 by mass spectrometry in ovarian cancer cells. We used co-immunoprecipitation assays and GST-pulldown to identify their interaction and found out the interaction site is located after the 26^th^ amino acid at the N terminus of ANXA2. The expression of ANXA2 and HE4 were up-regulated simultaneously and their overexpression promoted the malignant behavior of ovarian cancer [24, 25]. Researches showed that, the expression of ANXA2 and HE4 were up-regulated in endometrial carcinoma, respectively [26, 27]. In 2014, Alonso et al. [26] reported that detection of recurrent endometrial carcinoma could be achieved by comparing the level of ANXA2 expression between recurrent and primary lesions. It was also reported that serum HE4 can be used as a prognostic marker and pre-operative risk evaluation index in endometrial carcinoma patients [27]. Does the interaction between ANXA2 and HE4 exist in endometrial carcinoma and promote its malignant biological behavior? The two molecules are considered to be closely related to the tumorigenesis and progression of malignant tumors. However, studies so far have not tested the expression of ANXA2 and HE4 in endometrial carcinoma, simultaneously. So does the interaction of them in endometrial carcinoma. In the current study, for the first time, we simultaneously compared the expression level of ANXA2 and HE4 in endometrial tissues by immunohistochemistry assays, analyzed the correlation of the two molecules and investigated the effects of them on the development of endometrial carcinoma, and the interaction between ANXA2 and HE4 in the progression of endometrial carcinoma. We hope this study will contribute to the diagnosis, treatment and prognosis assessment of endometrial caicinoma.

## Materials and methods

### Patients and paraffin-embedded tissue samples

The study was approved by the Research Ethic Board at Shengjing Hospital affiliated to China Medical University. Our study population consisted of 132 patients and the specimens were collected during operation from the Department of Obstetrics and Gynecology of Shengjing Hospital affiliated to China Medical University between 2004 and 2013. The paraffin-fixed pathologic specimens had histopathologic-confirmed diagnoses by in-house experts, as follows: endometrial carcinoma, *n* = 84; atypical hyperplasia of endometrium (mild hyperplasia, *n* = 8; moderate hyperplasia, *n* = 9; and severe hyperplasia, *n* = 13), *n* = 30; and endometrium (secretory and proliferative phase, *n* = 9 each), *n* = 18 (Table 1). The normal endometrium specimens were collected from patients with undesired fertility who had vaginal hysterectomies of the entire uterus or entire uterus and bilateral adnexa due to cervical lesions, with no uterine myomas, ovarian chocolate cysts, or other estrogen-dependent diseases in the atypical hyperplasia and normal endometrium groups. Patients in the endometrial carcinoma group were 36–79 years of age with an average age of 58.93 years, patients in the endometrial atypical hyperplasia group were 30–66 years of age with an average age of 45.30 years, and patients in the normal endometrium group were 39–53 years of age with an average age of 44.06 years; no statistically significant difference existed among the average ages of each group (*p* > 0.05). All of the patients had primary endometrial carcinomas with complete clinical and pathologic data, and no patients received pre-operative chemotherapy and/or hormone therapy (clinicopathological parameters seen in Table 2).

**Table 1:** Expression of ANXA2 and HE4 in different endometrial tissues

Groups	Cases	ANXA2					HE4				
		-	+	++	+++	Positive (%)	-	+	++	+++	Positive (%)
Malignant	84	4	25	27	28	95.2 %^a^	12	34	25	13	85.7 %^c,d^
Atypical	30	5	9	7	9	83.3 %^b^	10	13	7	0	66.7 %^e^
Severe	13	2	3	3	5	84.6 %	3	5	5	0	76.9 %
Moderate	9	1	3	3	2	88.9 %	2	5	2	0	77.8 %
Mild	8	2	3	1	2	75.0 %	5	3	0	0	37.5 %
Normal	18	8	2	1	7	55.6 %	15	3	0	0	16.7 %
Proliferative	9	5	1	0	3	44.4 %	8	1	0	0	11.1 %
Secretory	9	3	1	1	4	66.7 %	7	2	0	0	22.2 %

^a,b^positive ANXA2 cases in malignant and atypical, respectively, compared with normal group, both *p* < 0.05 (*p*_a_ < 0.001, *p*_b_ = 0.049)

^c,d^positive HE4 cases in malignant compared with atypical and normal group, respectively, both *p* < 0.05 (*p*_c_ = 0.023, *p*_d_ < 0.001)

^e^positive HE4 cases in atypical group campared with normal group, *p* < 0.05 (*p*_e_ = 0.001)

**Table 2:** Relationships between the expression of ANXA2, HE4 and clinicopathological parameters of 84 endometrial carcinoma patients

Parameters	Cases	ANXA2				HE4			
		Positive exp. (%)	*P*-value	High exp. (%)	*P*-value	Positive exp. (%)	*P*-value	High exp. (%)	*P*-value
Age at diagnosis									
< 59	42	39(92.9 %)	*p* > 0.05	26(61.9 %)	*p* > 0.05	36(85.7 %)	*p* > 0.05	18(42.9 %)	*p* > 0.05
≥ 59	42	41(97.6 %)		29(69.0 %)		36(85.7 %)		20(47.6 %)	
FIGO stage									
I	53	49(92.5 %)	*p* > 0.05	29(54.7 %)	*p*_*I-II/III-IV*_ = 0.001	45(84.9 %)	*p* > 0.05	20(37.7 %)	*p*_*I-II/III-IV*_ = 0.013
II	7	7(100 %)		4(57.1 %)		5(71.4 %)		2(28.6 %)	
III	21	21(100 %)		19(90.5 %)		19(90.5 %)		13(61.9 %)	
IV	3	3(100 %)		3(100 %)		3(100.0 %)		3(100 %)	
Pathologic type									
Endometiod	39	35(89.7 %)	*p* > 0.05	23(59.0 %)	*p* > 0.05	34(87.2 %)	*p* > 0.05	18(46.2 %)	*p* > 0.05
Serous	19	19(100 %)		13(68.4 %)		15(78.9 %)		6(31.6 %)	
Clear cell	17	17(100 %)		12(70.6 %)		16(94.1 %)		8(47.1 %)	
Others^a^	9	9(100 %)		7(77.8 %)		7(77.8 %)		6(66.7 %)	
Differentiation									
Well	17	14(82.4 %)	*p*_*well/poor*_ = 0.020	4(23.5 %)	*p*_well/mod._ = 0.003	13(76.5 %)	*p* > 0.05	3(17.6 %)	*p*_well/mod._ = 0.009
Moderate	24	23(95.8 %)		17(70.8 %)	*p*_*well/poor*_ < 0.001	22(91.7 %)		14(58.3 %)	*p*_well/poor_ = 0.026
Poor	43	43(100 %)		34(79.1 %)		37(86.0 %)		21(48.8 %)	
ER^b^									
-	45	44(97.8 %)	*p* > 0.05	33(73.3 %)	*p*_−/+_ = 0.031	40(88.9 %)	*p* > 0.05	21(46.7 %)	*p* > 0.05
+	27	24(88.9 %)		13(48.1 %)		22(81.5 %)		11(40.7 %)	
Unknown	12	12(100 %)		9(75.0 %)		10(83.3 %)		6(50.0 %)	
PR^c^									
-	43	43(100 %)	*p*_*−/+*_ = 0.023	33(76.7 %)	*p*_−/+_ = 0.006	39(90.7 %)	*p* > 0.05	21(48.8 %)	*p* > 0.05
+	29	25(86.2 %)		13(44.8 %)		24(82.8 %)		12(41.4 %)	
Unknown	12	12(100 %)		9(75.0 %)		9(75.0 %)		5(41.7 %)	
Muscular invasion									
< 1/2	51	47(92.2 %)	*p* > 0.05	28(54.9 %)	*p* = 0.011	42(82.4 %)	*p* > 0.05	17(33.3 %)	*p* = 0.006
≥ 1/2	33	33(100 %)		27(81.8 %)		30(90.9 %)		21(63.6 %)	
LN metastasis^d^									
-	50	48(96.0 %)	*p* > 0.05	29(58.0 %)	*p*_−/+_ = 0.003	44(88.0 %)	*p* > 0.05	18(36.0 %)	*p*_−/+_ = 0.016
+	19	19(100 %)		18(94.7 %)		18(94.7 %)		13(68.4 %)	
Unknown	15	13(86.7 %)		8(53.3 %)		10(66.7 %)		7(46.7 %)	

^a^”Others” including 4 mucous carcinoma, 2 squamous carcinoma, 2 undifferentiated carcinoma, 1 small cell carcinoma

^b^12 patients without ER detection

^c^12 patients without PR detection

^d^15 patients without lymphadenectomy

### Cell culture

An ovarian cancer cell line (CaoV-3) and endometrial carcinoma cell lines (HEC-1A and Ishikawa) were purchased from Cell Culture Collection of Shanghai and propagated in McCoy’s 5A modified medium with 10 % fetal bovine serum. Cell culture was according to the manufacturer’s protocol. All the cell lines were grown at 37 °C in a 5 % CO_2_/95 % air atmosphere and were revived every 3 to 4 months.

### Immunohistochemistry and immnocytochemistry staining

The endometrial tissue specimens were dissected using 5-μm serial consecutive sections. Immunohistochemical staining was carried out as previously described [24]. Each tissue had two serial sections. Expression patterns of HE4 and ANXA2 in ovarian carcinoma tissues were analyzed *via* immunohistochemical streptavidinperoxidase staining. Positive and negative immunohistochemistry controls were routinely employed. Normal epididymis tissue served as a positive control for HE4, while breast cancer tissue was used as the positive control for ANXA2 antigen. The negative control was incubated with rabbit IgG (Bioss, China) instead of primary antibody. The working concentrations of primary antibodies against HE4 and ANXA2 used were 1:40 (Abcam, Rabbit polyclonal to HE4) and 1:1200 (Abcam, Rabbit polyclonal to ANXA2), respectively. The empirical procedure was performed based on the manufacturer's instructions.

Cells at exponential phase of growth were digested by 0.25 % trypsin and cultured in medium containing 10 % FBS. Adherent cells with 30-40 % confluence were washed twice with cold PBS when growing in a single layer, and fixed with 4 % para- formaldehyde for 30 min. The rest steps were the same as immunohistochemistry.

We consider a positive result if there are buffy granules in the cell membrane and cytoplasm. According to the chromatosis intensity, no pigmentation, light yellow, buffy, and brown are scored 0, 1, 2, and 3, respectively. We choose 5 high-power fields in series from each slice, then score them and take the mean percentage of the chromatosis cells: chromatosis cells that account less than 5 % are 0, 5 % to 25 %: 1, 26 % to 50 %: 2, 51 % to 75 %: 3, and greater than 75 %: 4. Multiply these 2 numbers: 0 to 2 is considered (−); 3 to 4, (+); 5 to 8, (++); and 9 to 12, (+++). The scoring and corresponding images were presented (Additional file 1: Figure S1). Two pathologists read the sections to control error, independently.

### Co-immunoprecipitation and Western Blot

Ice-cold RIPA buffer (1 ml) was added to the endometrial carcinoma cells and ovarian cancer cells, followed by incubation for 30 min at 4 °C. After centrifugation at 15,000 × g for 30 min at 4 °C, the supernatant was collected and treated with 2 μg of mouse anti-ANXA2 monoclonal (Proteintech, Chicago, America) or goat anti-HE4 polyclonal antibody (Santa Cruz Biotechnology, Inc., Santa Cruz, CA, USA) for 3 h at 4 °C. Then, 20 μl of protein A/G PLUS-Agarose (Santa Cruz Biotechnology, Inc.) was added, followed by incubation on a rocker platform overnight at 4 °C. The primary antibody was replaced by mouse or goat IgG (Bioss, China) as negative control. Immunoprecipitates were subsequently subjected to 12 % SDS gel electrophoresis and analyzed *via* western blot using rabbit HE4 monoclonal (Abcam) and mouse ANXA2 monoclonal antibodies (Proteintech). Proteins were visualized using ECL reagent (Thermo scientific ECL). The experiments were repeated three times.

### Double-labeling immunofluorescence method

The endometrial tissue sections displayed positive expression of ANXA2 and HE4 in immunohistochemistry and endometrial carcinoma cell lines HEC-1A and Ishikawa were selected for the double-labeling immunofluorescence method. The sections and cells were simultaneously incubated with primary antibodies against HE4 (1:50 [rabbit]; Abcam) and ANXA2 (1:50 [mouse]; Proteintech). The primary antibody was replaced by rabbit or mouse IgG for negative controls. The working concentrations of fluorescein isothiocyanate (FITC) and tetraethyl rhodamine isothiocyanate (TRITC) were 1:50. Nuclei were counterstained with 4′,6-diamidino-2-phenylindole (DAPI). The empirical procedure was performed according to the manufacturer's instructions.

## Statistical analysis

The SPSS17.0 software system (SPSS, Inc., Chicago, IL, USA) was used for statistical analysis. *χ*^2^ analysis, variance analysis and *t*-test were employed. Cox regression model was used for analysis of risk factors. Kaplan–Meier and log-rank methods were used to analyze and compare survival curves. Spearman correlation analysis and regression model were used to analyze the correlation between the two proteins. A bilateral *p* < 0.05 was considered statistically significant.

## Results

### Co-expression of ANXA2 and HE4 in endmetrial cancer tissue sections and cells

**Fig. 1:**
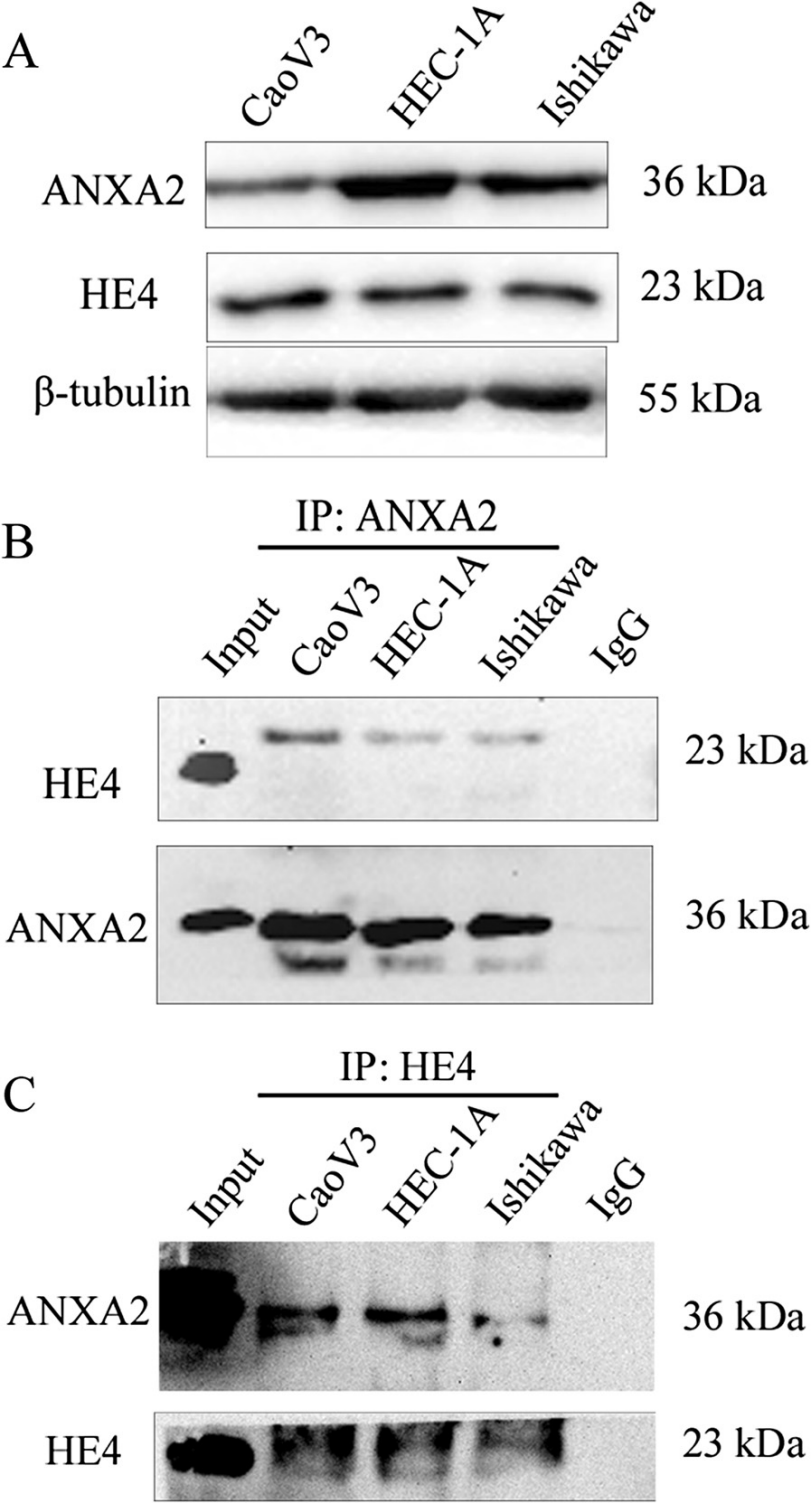
Expression and interaction of ANXA2 and HE4 in ovarian and endometrial cancer cells. Legends: Western blot detected the expression of ANXA2 and HE4 in ovarian cancer and endometrial cancer cell lines (CaoV3, HEC-1A, Ishikawa) (**A**). Cell lyste from CaoV3 and Ishikawa and HEC-1A cell lines was immunoprecipitated with anti-ANXA2 antibody (**B**) and anti-HE4 antibidy (**C**) and then immunoblotted with anti-HE4 antibody and anti-ANXA2 antibody, “IgG” representing the negtive control. “Input” was total cell lysate of CaoV3

**Fig. 2:**
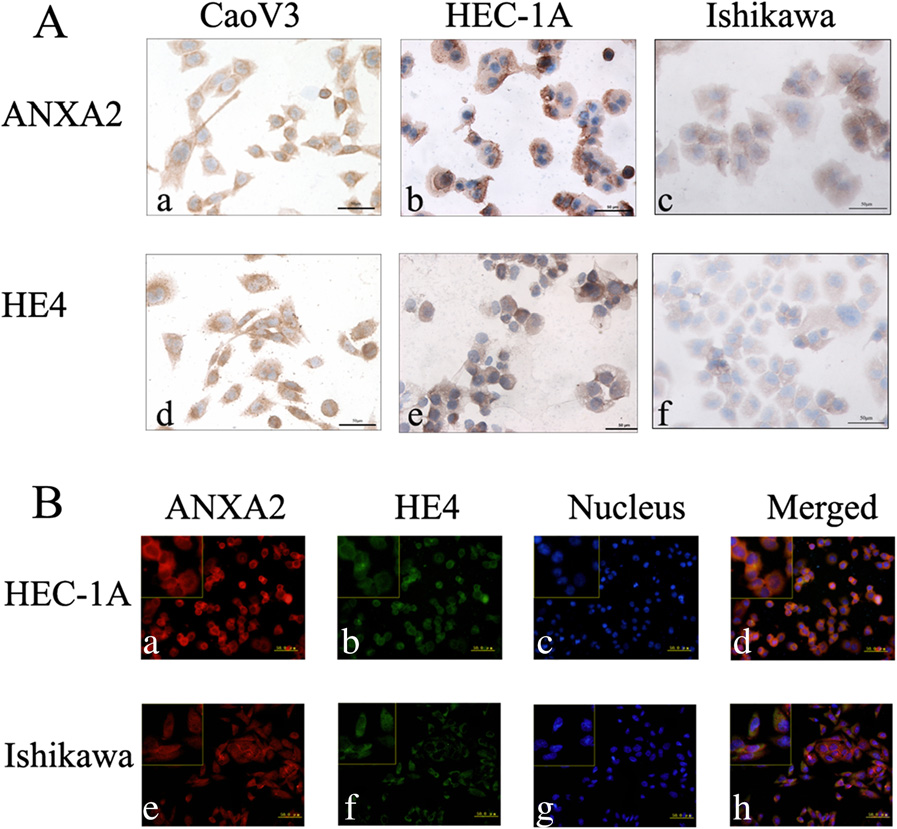
Expression and co-localization of ANXA2 and HE4 in cancer cells. Legends: **A** Expression of ANXA2 and HE4 in ovarian cancer cells CaoV3 (a, d) and endometrial camcer cells HEC-1A (b, e), Ishikawa (c, f), detected by immunocytochemistry. **B** Double-labeling immunofluoscence showed the colocalization of HE4 and ANXA2 in HEC-1A (a ~ d) and Ishikawa (e ~ h). The color of “red” represents ANXA2; “green” represents HE4; “blue” represents nucleus; “orange” represents the colocalization of ANXA2 and HE4. The scale ruler represents 50 μm; picture magnification is 400 × (**A**, **B**) and then magnified twice in the small box at the top-left corner (**B**)

**Fig. 3:**
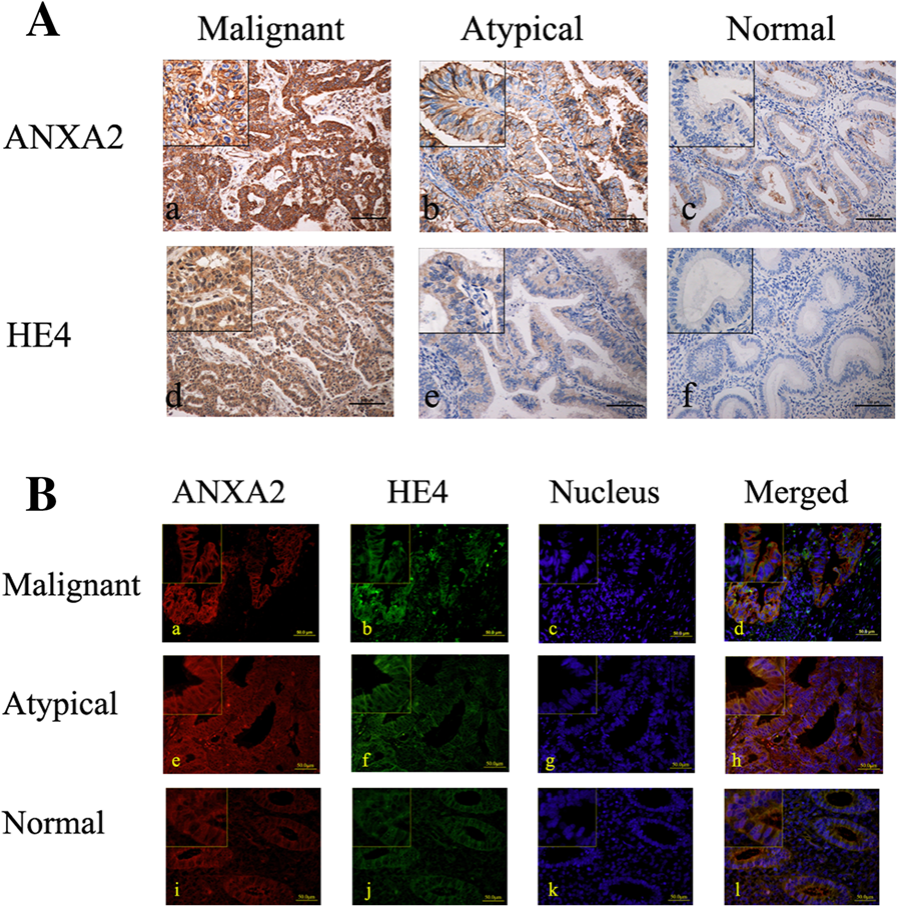
Expression and co-localization of ANXA2-HE4 in endometrial tissues. Legends: **A** Immunohistochemical micrographs of ANXA2 and HE4 in malignant tissues (a, d), atypical tissues (b, e), normal tissues (c, f); The scale ruler represents 100 μm; picture magnification is 200× and 400× for the small box at the top-left corner. **B** Colocalization of ANXA2 and HE4 detected by double-labeling immunofluoscence in malignant tissues (a ~ d), atypical tissues (e ~ h), and normal tissues (i ~ l). The color of “red” represents ANXA2; “green” represents HE4; “blue” represents nucleus; “orange” represents the colocalization of ANXA2 and HE4. The scale ruler represents 50 μm; picture magnification is 400× and then magnified twice in the small box at the top-left corner

Results of western blot and immunocytochemical testing confirmed the expression of ANXA2 and HE4 in endometrial carcinoma cell lines (Ishikawa and HEC-1A; Figs. 1A and 2A). Then, co-immunoprecipitation testing detected the interaction between ANXA2 and HE4 in the two cell lines (Fig. 1B and C). Immunofluorescence revealed co-expression of ANXA2 labeled by red fluorescence and HE4 labeled by green fluorescence in the cell membrane and cytoplasm of endometrial carcinoma cell lines (Fig. 2B) and different endometrial tissues (Fig. 3B), and the overlapping orange fluorescence observed at the expression sites suggested co-localization of ANXA2 and HE4.

### Expression of ANXA2 and HE4 in different endometrial tissues

ANXA2 and HE4 coloration mainly occurred in the cell membrane, which was also observed in the cytoplasm (Fig. 3A). The rate of positive expression of ANXA2 in endometrial carcinoma, atypical hyperplasia, and normal endometrium tissues was 95.2 % (80/84), 83.3 % (25/30), and 55.6 % (10/18), respectively (Table 1). The rates of positive expression in malignant and atypical hyperplasia tissues were significantly higher than the rates of positive expression in normal tissues (both *p* < 0.05) (Table 1). The positive rates of expression of ANXA2 in the moderate and severe atypical hyperplasia groups were 88.9 % (8/9) and 84.6 % (11/13), respectively, which were higher than the mild group (75.0 % [6/8]; both *p* > 0.05) (Table 1). The positive rate of expression of secretory phase endometrium was 66.7 % (6/9), which was higher than proliferative endometrium (44.4 % [4/9]; *p* > 0.05) (Table 1).

The positive rate of expression of HE4 in the endometrial carcinoma group was 85.7 % (72/84), which was significantly higher than the atypical hyperplasia 66.7 % (20/30) and normal control groups 16.7 % (3/18; both *p* < 0.05) (Table 1). The positive rate of expression of HE4 in atypical hyperplasia was also significantly higher than normal control groups (*p* < 0.05). The positive rates of expression of HE4 in the moderate and severe atypical hyperplasia groups were 77.8 % (7/9) and 76.9 % (10/13), respectively, which were higher than the mild atypical hyperplasia group (37.5 % [3/8]; both *p* > 0.05) (Table 1). The positive rate of expression in secretory phase endometrium was 22.2 % (2/9), which was slightly higher than the proliferative phase endometrium (11.1 % [1/9]; *p* > 0.05) (Table 1).

### Relationship between the expression of ANXA2/HE4 and the clinicopathologic parameters of endometrial carcinoma

The study included 84 cases of endometrial carcinoma with positive expression of ANXA2 in well, moderately, and poorly differentiated groups (82.4 % [14/17], 95.8 % [23/24], and 100 % [43/43], respectively), which increased with the reduction in the degree of differentiation (Table 2). The positive rate of expression of ANXA2 in the well differentiated group was significantly lower than the poorly differentiated group (*p* < 0.05) (Table 2). The expression rate of ANXA2 in the PR-negative group (100 % [43/43]) was significantly higher than the PR-positive group (86.2 % [25/29], *p* < 0.05) (Table 2). When the cases of endometrial carcinoma were further divided into high (++/+++) and low ANXA2 expression groups (−/+), the high expression rate of ANXA2 in stage III–IV endometrial carcinoma patients was 91.7 % (22/24), which was significantly higher than stage I–II patients (55.0 % [33/60], *p* < 0.05) (Table 2). With a reduction in the degree of differentiation, the high expression rate of ANXA2 increased gradually; specifically, the high expression rate in moderately and poorly differentiated groups were 70.8 % (17/24) and 79.1 % (34/43), respectively, which were significantly higher than the well differentiation group (23.5 % [4/17]; both *p* < 0.05) (Table 2). High expression rate of ANXA2 in the ER- and PR-negative groups were 73.3 % (33/45) and 76.7 % (33/43), respectively, which were significantly higher than the ER- and PR-positive groups (48.1 % [13/27] and 44.8 % [13/29], respectively; both *p* < 0.05) (Table 2). The high expression rate of ANXA2 in the deep myometrial invasion group was 81.8 % (27/33), which was significantly higher than the superficial myometrial invasion group (54.9 % [28/51]; *p* < 0.05) (Table 2). The high expression rate of ANXA2 in the lymph node metastasis group was 94.7 % (18/19), which was significantly higher than the negative lymph node metastasis group (58.0 % [29/50]; *p* < 0.05) (Table 2). No significant difference in the expression of ANXA2 was detected among the different pathologic tumor-types (*p* > 0.05) (Table 2).

Similar to ANXA2, the high expression rate of HE4 in stage III–IV endometrial carcinoma was 66.7 % (16/24), which was significantly higher than stage I–II (36.7 % [22/60]; *p* < 0.05) (Table 2). The high expression rate of HE4 in moderately and poorly differentiated carcinoma was 58.3 % (14/24) and 48.8 % (21/43), respectively, which was significantly higher than well-differentiated cancer (17.6 % [3/17]; both *p* < 0.05) (Table 2). The high expression rate of HE4 in the positive lymph node metastasis group was 68.4 % (13/19), which was significantly higher than the negative lymph node metastasis group (36.0 % [18/50]; *p* < 0.05) (Table 2). The high expression rate of HE4 in the deep muscular layer invasion group was 63.6 % (21/33), which was significantly higher than the superficial myometrial invasion group (33.3 % [17/51]; *p* < 0.05) (Table 2). No significant difference in the expression of HE4 was detected among the different pathologic tumor-types, and ER and PR expression (*p* > 0.05) (Table 2).

### ANXA2 and HE4 overexpression in endometrial carcinoma predicts patient survival

Until November 2014, with a follow-up time of 16–126 months, among the 84 patients with endometrial carcinoma, 19 died of recurrent cancer, 3 survived with tumor recurrences, 43 survived without tumor recurrence, and the other 19 were lost to follow-up. The mortality and recurrence rates of the ANXA2 and HE4 high expression group were 42.9 % (18/42) and 47.6 % (20/42), and 44.8 % (13/29) and 51.7 % (15/29), respectively, which were significantly higher than the low expression group (4.3 % [1/23] and 8.7 % [2/23], and 16.7 % [6/36] and 19.4 % [7/36], respectively; all *p* < 0.05) (Additional file 2: Table S1).

**Fig. 4:**
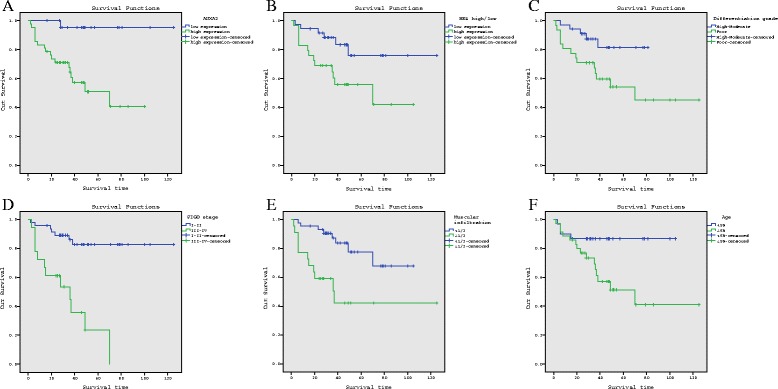
Univariate Kaplan–Meier analysis of endometrial cancinoma. Legends: The blue line means patients with low ANXA2 expression (**A**) low HE4 expression (**B**) well-moderate differentiated (**C**) I ~ II surgical stage (**D**) superficial muscular infiltration (**E**) and yonger patients (**F**) showed significantly longer overall survival

Univariate Kaplan–Meier analysis revealed that high expression of ANXA2 and HE4 were significantly correlated with a shortened overall survival (both *p* < 0.05) (Fig. 4 and Table 3). The average survival time of the ANXA2 and HE4 high expression group was 35.40 and 37.14 months, while the average survival time of the low expression group was 50.26 and 43.50 months, respectively. In addition, age (<59 years *vs.* ≥ 59 years), FIGO staging (I–II *vs.* III–IV), degree of differentiation (well - moderate *vs.* poor), and depth of myometrial invasion (<1/2 *vs.* > 1/2) were all associated with a poor prognosis (all *p* < 0.05) (Fig. 4 and Table 3).

**Table 3:** Survival analysis and prognosis analysis of endometrial carcinoma. Univariate Kaplan-Meier analysis of the prognosis of endometrial carcinoma

Variable	Characteristics	(Log-rank) *p*-value
Age at diagnosis	<59y *vs.* ≥59y	0.020
FIGO stage	I-II *vs.* III-IV	<0.001
Differentiation grade	Well-mod. *vs.* poor	0.027
Muscular invasion	<1/2 *vs.* ≥1/2	0.002
ANXA2	Low *vs.* high	0.002
HE4	Low *vs.* high	0.018
Multivariate Cox regression analysis of patients with endometrial carcinoma		
Variables	*p*-value	Hazard ratio (95 % CI)
FIGO stage (I-II vs III-IV)	0.002	4.593 (1.763-11.964)
ANXA2 (low vs high)	0.046	8.004 (1.036-61.844)

Based on univariate analysis, the multivariate Cox regression model was used for the analysis of prognostic risk factors; the results are listed in Table 4. Age at diagnosis, FIGO stage, differentiation, depth of invasion, and the level of expression of ANXA2 and HE4 were included in the analysis model. Multivariate Cox regression analysis showed that overexpression of ANXA2 and advanced FIGO staging were independent risk factors for the prognosis of endometrial carcinoma (Table 3).

**Table 4:** The correlation between ANXA2 and HE4 expression in endometrial carcinoma. (the Spearman correlation coefficient *r*_s_ was 0.228, *p* =0.037)

ANXA2	Cases	HE4	
		Negative	Positive
Negative	4	2	2
Positive	80	10	70
Cases	84	12	72

### Correlation between the expression of ANXA2 and HE4

There were 2, 2, 10, and 70 patients in the ANXA2-/HE4-, ANXA2-/HE4+, ANXA2+/HE4-, and ANXA2+/HE4+ groups, respectively. Correlation analysis showed that there was a positive correlation between the expression of ANXA2 and HE4 in endometrial carcinoma (Spearman correlation coefficient R_S_ = 0.228, *p* = 0.037) (Table 4).

Univariate linear regression analysis showed that the immunoactivity of ANXA2 and HE4 can affect each other (both *p* < 0.05) (Table 5). As was presented in Table 2, FIGO stage, differentiation degree, ER expression, PR expression, muscular invasion degree and lymph nodes metastasis were significant affective factors of ANXA2 expression. In multivariate linear regression analysis of ANXA2 expression score (from 0 to 12), the results showed that HE4 expression and differentiation degree or muscular invasion were independent factors of ANXA2 expression. Multivariate analysis showed muscular invasion was independent factors of HE4 expression (Table 5).

**Table 5:** Linear regression analysis of ANXA2 and HE4 expression

	ANXA2 score				HE4 score			
	Univariate		Multivariate		Univariate		Multivariate	
	β	*p*	β	*p*	β	*p*	β	*p*
HE4 score	0.267	0.014	0.251	0.018^b^				
ANXA2 score					0.268	0.014	0.202	0.064^c^
FIGO	0.828	0.020			0.601	0.096		
Differentiation	1.657	0.016	1.555	0.020^b^	0.409	0.559		
ER	−0.815	0.303			0.207	0.791		
PR	−1.665	0.031			0.011	0.988		
Muscular invasion	1.693	0.016	2.006	0.009^a^	1.959	0.005	1.959	0.005^c^
LN metastasis	1.521	0.074			1.387	0.111		

^a^represents the multivariate regression analysis when ANXA2 score was dependent variate, including HE4 score, FIGO, differentiation, PR and muscular invasion as independent variables

^b^represents the results of model 2 in multivariate regression analysis when excluding PR as independent variables

^c^represents the multivariate regression analysis when HE4 score was dependent variate, including ANXA2 score and muscular invasion as independent variables

## Discussion

ANXA2 is a calcium-dependent phospholipid-binding protein, widely existing on the cell surface membranes of tumor cells, endothelial cells, macrophages, and monocytes. ANXA2 is involved in membrane transport and a series of membrane surface calmodulin-dependent biological functional activities, including cell migration, inflammation, fibrinolysis, exocytosis, endocytosis, signal transduction, and cellular proliferation, differentiation, and apoptosis [15]. As a co-receptor of tissue plasminogen activator and plasminogen, ANXA2 promotes the formation and activation of plasmin and the downstream matrix metalloproteinases, and promotes extracellular matrix remodeling, angiogenesis, invasion, and metastasis of tumor cells. ANXA2 also interacted with P-gp and HAb18G/CD147 to promote malignant biological behavior of malignant tumors, such as drug resistance, proliferation, and adhesion. ANXA2 plays an important role in biological activity and tumor progression [18, 28, 29].

Recently, our team discovered another ANXA2 interacting protein, HE4, a new tumor marker. For the first time, HE4 received a lot of attention due to its highly specific expression in ovarian cancer [20]. Through mass spectrometry analysis, co-immunoprecipitation, and immunofluorescence, we confirmed the interaction between these two proteins in ovarian cancer cells, and found that the combination of ANXA2 and HE4 activated the MAPK and FOCAL signaling pathways to promote the invasion and migration of ovarian cancer cells [24].

This study examined the expression of ANXA2 and HE4 in endometrial carcinoma cells using western blot and immunocytochemistry, and confirmed their interaction in endometrial carcinoma cells through co-immunoprecipitation and double-labelling immunofluorescence. We further examined the expression levels of ANXA2 and HE4, and showed that the levels of ANXA2 expression in endometrial carcinoma and atypical hyperplasia were significantly higher than normal endometrium (Table 1), and high ANXA2 expression was related to lymph node metastasis and depth of myometrial invasion (Table 2). The level of HE4 expression in endometrial carcinoma was also significantly elevated, with a correlation detected between the expressions of these two proteins. Therefore, we hypothesized that ANXA2 interacted with HE4 to promote tumor invasion and metastasis in endometrial carcinoma. The results of our study showed that the ANXA2 and HE4 expression in stage III–IV endometrial carcinoma patients was higher than stage I–II patients (Table 2), which was correlated with FIGO staging and the prognosis of survival in patients with endometrial carcinoma (Table 3), as further supported by studies investigating the role of ANXA2 and HE4 in other tumors [30–33].

Currently, the studies investigating ANXA2 in endometrial tissues are limited, with only a few studies showing that ANXA2 might play an important role in endometriosis [34], adenomyosis angiogenesis [35], and embryo implantation [36, 37]. In 2009, Dominguez et al. [36] detected significantly different expression of ANXA2 during the implantation window by protein mass spectrometry analysis. The investigation further showed [37] inhibition of ANXA2 expression significantly eliminated embryo adhesion and reduced the activity of RhoA, and ANXA2 may regulate F-actin remodeling to affect the RhoA/ROCK signaling pathway, thus promoting human placenta adhesion. In 2012, Zhou et al. [35] reported that estrogen can lead to increased expression of ANXA2 in an *in vitro* adenomyosis model, and the increase in ANXA2 expression promoted endometrial epithelial mesenchymal transformation. At the same time, the increased expression of ANXA2 may promote the angiogenesis activity of adenomyosis endometrial cells through the HIF-1 alpha /VEGF-A pathway. In 2014, through proteomic and immunohistochemical analysis of recurrent and primary endometrial carcinoma, Alonso et al. [26] determined that ANXA2 can be used as a potential marker of endometrial carcinoma recurrence, and ANXA2 causes a relapse through the promotion of endometrial carcinoma metastasis, rather than its effect on the sensitivity of radiotherapy and chemotherapy; further knocking down of ANXA2 in a mouse model led to a decreased spread of tumor cells circulating in the blood; their retrospective studies have shown that ANXA2 can effectively predict the recurrence of endometrial carcinoma. Our research also showed that the recurrence rate in ANXA2 high expression group was significantly higher than ANXA2 low expression group (Additional file 2: Table S1). ANXA2 expression in endometrial carcinoma tissues can be used as an independent risk factor for prognosis; the prognostic risk in patients with high expression of ANXA2 was eight times higher than patients with low expression (Table 3).

This study is the first study to examine the relationship between the expression of ANXA2 in different endometrial tissues and endometrial carcinoma clinicopathologic parameters, and to analyze the correlation between the expression of ANXA2 and HE4, suggesting that ANXA2 and HE4 may play an important role in the prognosis of endometrial carcinoma. Furthermore, the current study found that in addition to ovarian cancer, ANXA2 and HE4 also interact in endometrial carcinoma, and co-localized in the cell membrane and cytoplasm.

## Conclusions

In summary, ANXA2 and HE4 were overexpressed in endometrial carcinoma. The expression levels of ANXA2 and HE4 were positive correlated and the two proteins played important roles in the process of endometrial malignant transformation. ANXA2 and HE4 can be used as biomarkers to evaluate the prognosis of endometrial carcinoma.

## Additional files

Additional file 1: Figure S1. Representative images of different classification. Legends: A. endometrioid adenocarcinoma, poor differentiation grade, FIGO stage III, scoring 3*4(ANXA2), 3*4(HE4); B. serous adenocarcinoma, poor differentiation grade, FIGO stage I, scoring 2*2(ANXA2), 2*3(HE4); C. clear cell carcinoma, poor differentiation grade, FIGO I, scoring 2.5*4(ANXA2), 2.5*4(HE4); D. undifferentiated carcinoma, poor differentiation grade, FIGO I, scoring 2*4(ANXA2),1.5*3(HE4); E. squamous cell carcinoma, moderate differentiation grade, FIGO I, scoring 3*3(ANXA2), 2*4(HE4); F. mucinous adenocarcinoma, poor differentiation grade, FIGO I, scoring 3*4(ANXA2), 1.5*3(HE4); G. small cell carcinoma, poor differentiation grade, FIGO I, scoring 3*3(ANXA2), 3*4(HE4). Magnification 200×, and 400× for top-left corner box. (TIFF 3007 kb) Additional file 2: Table S1. The correlation between ANXA2/HE4 and clinical outcomes of endometrial carcinoma. (DOC 37 kb)

## Abbreviations

ANXA2: Is short for Annexin A2
HE4: Is short for Human epididymis protein 4
HR: Is short for hazard ratio
FIGO: Is short for International Federation of Gynecology and Obstetrics
ER: Is short for estrogen receptor
PR: Is short for progestrone receptor
PBS: Is short for phosphate buffer
CA125: Is short for carbohydrate antigen 125
LN: Is short for lymph node
