# Supplementary figures and images for: Expression and clinical significance of annexin A2 and human epididymis protein 4 in endometrial carcinoma
Source: J Exp Clin Cancer Res. 2015 Sep 11;34(1):96. doi: 10.1186/s13046-015-0208-8 (PMC4567805; doi:10.1186/s13046-015-0208-8)

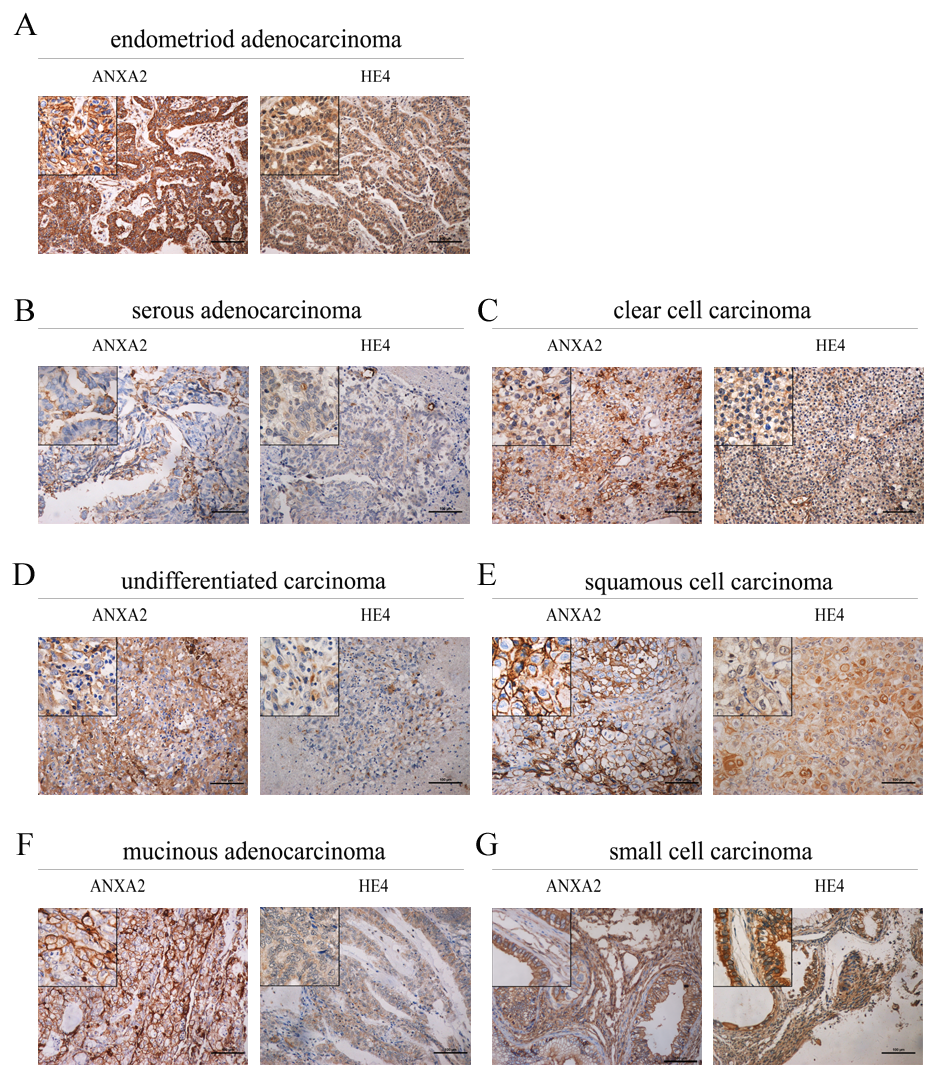

Supplement: Additional file 1: Figure S1. — Representative images of different classification. Legends: A. endometrioid adenocarcinoma, poor differentiation grade, FIGO stage III, scoring 3*4(ANXA2), 3*4(HE4); B. serous adenocarcinoma, poor differentiation grade, FIGO stage I, scoring 2*2(ANXA2), 2*3(HE4); C. clear cell carcinoma, poor differentiation grade, FIGO I, scoring 2.5*4(ANXA2), 2.5*4(HE4); D. undifferentiated carcinoma, poor differentiation grade, FIGO I, scoring 2*4(ANXA2),1.5*3(HE4); E. squamous cell carcinoma, moderate differentiation grade, FIGO I, scoring 3*3(ANXA2), 2*4(HE4); F. mucinous adenocarcinoma, poor differentiation grade, FIGO I, scoring 3*4(ANXA2), 1.5*3(HE4); G. small cell carcinoma, poor differentiation grade, FIGO I, scoring 3*3(ANXA2), 3*4(HE4). Magnification 200×, and 400× for top-left corner box. (TIFF 3007 kb) [file 13046_2015_208_MOESM1_ESM.tif]
