# Supplementary material for: Expression and clinical significance of annexin A2 and human epididymis protein 4 in endometrial carcinoma
Source: J Exp Clin Cancer Res. 2015 Sep 11;34(1):96. doi: 10.1186/s13046-015-0208-8 (PMC4567805; doi:10.1186/s13046-015-0208-8)
Supplement: Additional file 2: Table S1. — The correlation between ANXA2/HE4 and clinical outcomes of endometrial carcinoma. (DOC 37 kb) [file 13046_2015_208_MOESM2_ESM.doc]

**Supplementary Table S1**. The correlation between ANXA2/HE4 and clinical outcomes of endometrial carcinoma.

| Outcomes | Cases | ANXA2 | | | HE4 | | |
| --- | --- | --- | --- | --- | --- | --- | --- |
|  |  | Low | High | ***p*-value** | Low | High | ***p*-value** |
| Dead |  |  |  |  |  |  |  |
| Yes | 19 | 1 | 18 | ***p*=0.001** | 6 | 13 | ***p*=0.013** |
| No | 46 | 22 | 24 |  | 30 | 16 |  |
| Recurrence |  |  |  |  |  |  |  |
| Yes | 22 | 2 | 20 | ***p*=0.002** | 7 | 15 | ***p*=0.006** |
| No | 43 | 21 | 22 |  | 29 | 14 |  |
